# Characterization of Naturally Occurring NS5A and NS5B Polymorphisms in Patients Infected with HCV Genotype 3a Treated with Direct-Acting Antiviral Agents

**DOI:** 10.3390/v9080212

**Published:** 2017-08-07

**Authors:** Barbara Bartolini, Emanuela Giombini, Chiara Taibi, Raffaella Lionetti, Marzia Montalbano, Ubaldo Visco-Comandini, Gianpiero D’Offizi, Maria Rosaria Capobianchi, Fiona McPhee, Anna Rosa Garbuglia

**Affiliations:** 1 National Institute for Infectious Diseases “L. Spallanzani”, Via Portuense 292, 00149 Rome, Italy; barbara.bartolini@inmi.it (B.B.); emanuela.giombini@inmi.it (E.G.); chiara.taibi@inmi.it (C.T.); Raffaella.lionetti@inmi.it (R.L.); Marzia.montalbano@inmi.it (M.M.); Ubaldo.viscocomandini@inmi.it (U.V.-C.); Gianpiero.doffizi@inmi.it (G.D.); maria.capobianchi@inmi.it (M.R.C.); 2 Bristol-Myers Squibb Research and Development, Wallingford, CT 06492, USA; Fiona.Mcphee@bms.com; 3 Laboratory of Virology, National Institute for Infectious Diseases “L. Spallanzani”, Via Portuense 292, 00149 Rome, Italy

**Keywords:** HCV, genotype3, genetic variability, NS5A, NS5B, polymorphism, resistance-associated substitutions, ultra-deep pyrosequencing

## Abstract

Hepatitis C virus (HCV) genotype (GT)3 is associated with increased risk of steatosis, development of cirrhosis and hepatocellular carcinoma. Limited data are available regarding genetic variability and use of direct-acting antiviral agents in these patients. non-structural protein 5A (NS5A) and non-structural protein 5B (NS5B) sequencing was performed on 45 HCV GT3-infected Italian patients subsequently treated with sofosbuvir ± daclatasvir (SOF ± DCV). Novel GT3a polymorphisms were observed by Sanger sequencing in three NS5A (T79S, T107K, and T107S) and three NS5B (G166R, Q180K, and C274W) baseline sequences in patients who achieved sustained virological response (SVR). Baseline NS5A resistance-associated substitutions (RASs) A30K and Y93H were detected in 9.5% of patients; one patient with A30K did not achieve SVR. Phylogenetic analyses of sequences showed no distinct clustering. Genetic heterogeneity of NS5A and NS5B was evaluated using ultra-deep pyrosequencing (UDPS) in samples longitudinally collected in patients not achieving SVR. Some novel NS5A and NS5B polymorphisms detected at baseline may not impact treatment outcome, as they were not enriched in post-failure samples. In contrast, the clinically novel GT3 NS5A-L31F RAS emerged in one treatment failure, and I184T, G188D and N310S, located on the same NS5B haplotype, became predominant after failure. These findings suggest a potential impact of these novel substitutions on the treatment outcome; however, their significance requires further investigation.

## 1. Introduction

Hepatitis C virus (HCV) has been classified into seven genotypes (GTs) by employing phylogenetic analysis of whole genomes [1]. HCV GT3 is the second most prevalent genotype worldwide after GT1, with approximately 54.3 million cases [2]. Clinically, GT3 infection is associated with an increased risk of hepatic steatosis, development of cirrhosis and hepatocellular carcinoma (HCC). Moreover, in a retrospective analysis of HCC occurrence in HCV positive patients, GT3 infection was a strong predictor of HCC development: 34% versus 17% in non-GT3 (*p* = 0.013) [3].

During the era of pegylated interferon (pegIFN) + ribavirin (RBV) therapy, GT3 was considered an “easy-to-treat” genotype, with high cure rates compared with other viral genotypes [4]; most failures were observed in cirrhotic patients [5]. In the era of direct-acting antivirals (DAA), a wide range of potent drugs are approved for HCV treatment, but only a few of them are effective against GT3: sofosbuvir (SOF), a nucleotide analog inhibitor of the non-structural protein 5B (NS5B) polymerase, daclatasvir (DCV), a pan-genotypic non-structural protein 5A (NS5A) inhibitor and, recently, velpatasvir, another NS5A inhibitor [6].

In GT3 patients with cirrhosis, SOF + DCV treatment for 12 weeks resulted in 63% rate of sustained virologic response (SVR), compared with 96% without cirrhosis [7]. Addition of RBV increased the SVR rate to 91% [8]. Different treatment durations may also enhance SVR rate depending on a patient’s cirrhosis status [6,9]. The relevance of baseline NS5A resistance-associated substitutions (RAS) or other polymorphisms on DAA efficacy is still not completely understood for GT3, although reduced SVR rates have been reported for GT3 patients with baseline NS5A-Y93H [8].

At least 10 GT3 subtypes have been identified [10], with GT3a being the most prevalent. GT3 is predominant in injection drug users [11], and drug injection is considered the main route of transmission. In Europe, GT3a is predominant in Northern countries (Denmark, Finland and United Kingdom) and accounts for approximately up to 50% of all HCV infections in Norway [12].

In this study, genetic variability of NS5A and NS5B in 45 GT3a Italian patients, who received SOF ± DCV treatment with/without pegIFN + RBV, was evaluated. In particular, the type and frequency of amino acid substitutions, as well as the DAA selective pressure on the targeted gene regions was assessed. In addition, the RAS dynamics was analyzed by ultra-deep pyrosequencing (UDPS) in patients not achieving SVR.

## 2. Materials and Methods

### 2.1. Samples and Patients

Plasma samples from 45 HCV GT3a-infected patients receiving DAA-based therapies were obtained from our clinical center in Italy. The use of patient samples was approved by the Ethics Committee of the National Institute for Infectious Diseases “L. Spallanzani” (statement n20/2015). Written informed consent was obtained from all patients.

All patients were DAA treatment-naïve. Baseline characteristics including HCV-RNA, age, coinfection with human immunodeficiency virus (HIV) and cirrhosis status are reported in Table 1. Diagnosis of liver cirrhosis was based on clinical or histological features or with non-invasive assessment by transient elastography (stiffness > 14 KPa).

Hepatitis B serum surface antigens (HBsAg), anti-hepatitis virus surface antibodies (anti-HBs), HIV sero-status, serum alanine aminotransferase (ALT) levels, serum total bilirubin level, and serum platelet counts were also recorded (data available upon request).

All patients received SOF-based treatment ± DCV (as detailed in Table S3). All treatment decisions were in accordance with regulatory agency requirements [13]; all patients were treatment compliant.

### 2.2. Study Assessments

HCV genotype or subtype was determined using the RealTime HCV Genotype II assay (Abbott Molecular, Abbott Park, IL, USA). Plasma HCV RNA was assessed using the Abbott real time HCV RNA assay (Abbott Molecular) with a lower limit of quantitation (LLOQ) and of detection (LLOD) of 12 IU/mL. Time points for virological monitoring were: between 4 weeks pre-treatment and baseline visits, on-treatment every month, and post-treatment every month to post-treatment week 24. A tolerance of one week was accepted in the intervals of post-treatment virological monitoring. On-treatment, virological response was defined as HCV RNA < LLOQ with target not detected (HCV RNA < LLOQTND). Post-treatment virological response was defined as HCV RNA < LLOQ with/without target detected (HCV RNA < LLOQTD/TND).

Virological failure was defined as relapse: any confirmed HCV RNA measurement ≥LLOQ during post-treatment follow-up subsequent to an on-treatment response <LLOQ without target RNA detected (<LLOQTND) at the end-of-treatment visit.

Resistance testing was performed on plasma samples at baseline and at virological rebound. Detailed analysis of RAS was performed by UDPS in three of four patients experiencing therapy failure for whom available samples had sufficient HCV RNA levels (see below).

### 2.3. Nucleic Acid Extraction and PCR Amplifications

Viral RNA was extracted from 600 µL plasma using QIASYMPHONY (Qiagen, Hilden, Germany) and reverse transcribed by Moloney Murine Leukemia Virus (M-MULV) reverse transcriptase (Thermo Fisher Scientific, Waltham, MA, USA) using random hexamers.

The substitutions were identified using the strain NZL1 (accession number D17763) as GT3a reference, according to Smith et al. [1].

The HCV NS5A and NS5B regions were amplified with nested PCR using GT3-specific primers for NS5A and pan-genotypic primers for NS5B, as described [14,15]. The resulting amplicons were 637 nt long for NS5A, spanning nt positions 6102–6738, and 629-nt long for NS5B, spanning 8037–8665 nt in D17763 GT3a reference genome.

The same amplicons were used for UDPS. In the second round PCR, the primers were conjugated with multiplex identifiers for sample barcoding and to adaptors for UDPS.

### 2.4. Sequencing

Sanger sequencing of second round amplicons was performed using the ABI PRISM 3100 genetic analyzer (Applied Biosystems, Foster City, CA, USA). The cutoff frequency for detecting variants with Sanger sequencing was around 15% [16].

Ultra-deep pyrosequencing was carried out with the 454 Life Sciences platform (GS-junior, Roche Diagnostics, Branford, CT, USA), according to the manufacturer’s instructions.

Ultra-deep pyrosequencing was performed on NS5A and NS5B regions from Pt42 and Pt43 at baseline (T0), as well as at the first (T1) and second (T2) treatment failure visits. For Pt45, UDPS was performed on the NS5B region at T0 and T1. For T1 and T2, the first sample with HCV RNA ≥10,000 IU/mL was used for UDPS to avoid target re-sampling bias [17].

### 2.5. Data Analysis

Sanger-derived sequences were aligned with MUSCLE software [18] and manually verified using BioEdit software (Version 7.2.5). Amino acid substitutions were detected and compared with the D17763 reference sequence. All sequences were trimmed to include the largest common region encompassing amino acid positions NS5A 1-119 and NS5B 155-322. The mean inter-patient diversity of both regions was calculated with MEGA6 [19]. Specific focus was placed on amino acid positions under selective pressure that were inferred by calculating the mutation rates (dN–dS) in available GT3a sequences (53 for NS5A and 233 for NS5B by November 2016) present in the Los Alamos HCV database [20]. Normalized dN-dS was calculated using the Single likelihood ancestor counting (SLAC) method implemented in Pond et al. [21]. The frequency and types of polymorphisms present in the database sequences were also established.

HCV sequences obtained by Sanger sequencing in this study can be accessed in the GenBank database (accession numbers KY474420-KY474510)**.**

For the analysis of UDPS results, all reads underwent a correction pipeline with in-house-developed scripts that excluded reads <450 nt in length or with a mean quality score <20. Moreover, the sequences were clustered with CD-HIT software [22], using a 96% identity cutoff; clusters of <5 reads, or whose components represented <1% of sequences in the respective clinical sample, were excluded as previously described [23]. Nucleotide diversity was calculated for the region covered by both forward and reverse reads (nt positions referred to D17763: 6274-6555 for NS5A and 8167-8523 for NS5B) using the Phylip DNADIST software [24] and the F84 matrix. For the intra-sample polymorphism analysis, a threshold of 1% was applied as described previously [25]. For substitutions demonstrating enrichment at virological failure, lower baseline frequencies (i.e., 0.7%) were considered.

The presence of haplotypes was observed using a script developed in-house.

### 2.6. Phylogenetic Analysis

Phylogenetic trees were built using sequences of study patient as well as HCV GT3a sequences from the Los Alamos database, for which sampling year and geographic location was reported. For the Sanger sequences, nucleotide mixtures observed in the electropherogram were reported when representing ≥15% of the sequence population. The best-fit model (K2 + G + I) was selected according to the Akaike Information Criterion and trees were reconstructed using the maximum likelihood method implemented in MEGA6 [19]. Bootstrap values were calculated using 500 bootstrap iterations.

## 3. Results

### 3.1. Baseline Characteristics

Baseline demographic characteristics of 45 HCV GT-3-infected Italian patients are summarized in Table 1. Median age was 56 years; the majority of them (*n* = 35, 78%) had cirrhosis; 58% were treatment-naïve and 42% had been previously treated with pegIFN + RBV. Males represented 84% of patients with a median age of 56 years, 74% were cirrhotic and 60% were treatment-naïve. All women (*n* = 7, 16%) were cirrhotic and had a median age of 54 years; 43% (*n* = 3) were treatment-naive. The median baseline HCV RNA level for all patients was 5.3 Log10 IU/mL (range 1.1–7.0). Forty patients completed the treatment and achieved SVR; one (Pt13) died just after the end of treatment (EOT); four patients (Pt42, Pt43, Pt44 and Pt45) did not achieve SVR and were retreated with a SOF-containing regimen. Specifically, Pt42, a transplanted patient, was first treated with SOF + DCV for 12 weeks and relapsed four weeks after EOT. He was retreated with SOF + DCV in association with RBV for 24 weeks, but relapsed eight weeks after EOT. Pt43, in waiting list for transplantation, was treated with SOF + DCV for 24 weeks, but relapsed four weeks after EOT. He was retreated with SOF + RBV for 48 weeks, and relapsed three weeks after EOT. Pt44 and Pt45 were treated with SOF + RBV for 24 weeks, but relapsed four weeks after EOT. They were retreated with SOF + DCV + RBV for 24 weeks and achieved SVR12 (Table 2).

In this study, T1 represents the sampling after the first failure, and T2 represents the sampling after the second failure (see Materials and Methods Section).

### 3.2. Genetic Variability at Baseline 

Sequences from 42 patients were available for analysis; the frequency of polymorphic sites, established by comparison with the GT3a reference sequence D17763 [1], is reported in Table 3.

In NS5A, 42 amino acid positions showed polymorphism, with 13 positions harboring multiple substitutions (Table 3). Three of these (T79S in Pt15, T107K in Pt15 and T107S in Pt3, Pt6, and Pt10) were not detected in the public databases. All patients (*n* = 3) with these polymorphisms achieved SVR.

NS5A polymorphisms reported to be associated with DCV resistance in GT3a include M28T, A30K, L31F/I/M, and Y93H [7,26]. M28L was detected in one virological failure (Pt44). This polymorphism is not thought to be a GT3 RAS, and is the reference amino acid in other genotypes (GT1b, GT2, GT4a and GT5). A30K was observed in 9.5% of patients at baseline (Pt14, Pt19, Pt28 and Pt42), while only the reference amino acid was observed at position 31. Y93H was detected at baseline in only one patient (Pt28) who also harbored A30K and achieved SVR.

The presence of NS5A polymorphisms at positions 24, 32, 58, 62 and 92, which can reduce susceptibility to some NS5A inhibitors in other genotypes [16,27], were evaluated, even though their impact in GT3a is unclear. NS5A-P58A/S/T was observed in three patients (Pt6, Pt10, and Pt 43). Serine at position 62 is the reference residue in the Food and Drug Administration (FDA) guidelines (S52 HCV strain, accession number GU814263), and was present in the majority (85.7%) of patients, including those (*n* = 4) who experienced SOF-based treatment failure. A62T (*n* = 5) and A62P (*n* = 1) were also detected.

The frequency of substitutions at NS5A positions not associated with DAA resistance was consistent with the frequency observed in the Los Alamos database (Table S1).

In NS5B, 32 amino acid positions had polymorphisms that differed from D17763, with eight positions harboring more than one polymorphism (Table 3).

Three substitutions (G166R in Pt35; Q180K in Pt16; C274W in Pt28) were not observed in available GT3 NS5B sequences in the Los Alamos database (Table S1B); all three patients with these polymorphisms achieved SVR. Polymorphisms at amino acid positions 159, 282, and 321, associated with SOF resistance in GT3 [28], were not detected. However, polymorphisms were observed at other amino acid positions previously associated with nucleotide-resistant HCV [29]. The NS5B polymorphism N244D was observed in five patients (Pt1, Pt27, Pt31, Pt32 and Pt37), R309Q was detected in one patient (Pt8), and N310D was detected in two patients (Pt31 and Pt32); all achieved SVR. The frequency of substitutions at NS5B positions not associated with DAA resistance was consistent with those reported in the public databases (i.e., for N307G 100% in the Los Alamos database vs. 97.6% in the study patients) (Table S2B).

**Figure 1:**
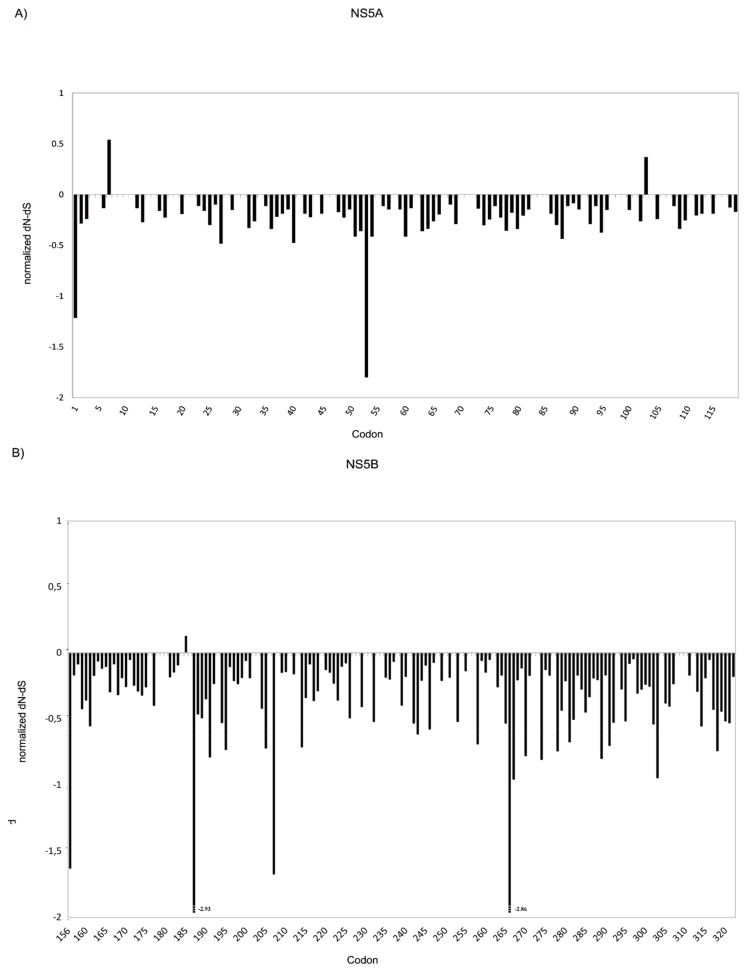
Substitutions rate at non-synonymous sites - substitutions rate at synonymous sites (dN-dS) in HCV GT3a sequences worldwide. The graphs represent dN-dS vs. each codon for: NS5A (**A**); and NS5B (**B**) of HCV GT3a sequences from the Los Alamos database.

NS5A and NS5B amino acid positions under drug selective pressure were evaluated by calculating the mutation rates (dN-dS) in GT3a sequences present in the Los Alamos HCV database [20]. For NS5A, results indicated that, among the positions involved in drug resistance, amino acids 24, 32, and 93 were associated with a negative selective pressure, while positions 28, 30, 31, 58, 62 and 92 appeared to be under neutral pressure (Figure 1A). Only two positions (7 and 103), not considered to be involved in NS5A inhibitor binding, hence to therapy response, were shown to be associated with a positive selective pressure in this analysis. In agreement with the public sequence database results, amino acid 7 was one of the more polymorphic sites in the baseline samples from the present study, with substitutions in 52.4% (22/42) of patients with available NS5A sequence.

For NS5B, results indicated that positions 159, 244, 282, 289, 316, 320, 321, involved in drug resistance, were under negative pressure while positions 309 and 310 appeared to be under neutral pressure (Figure 1B). The only amino acid position under positive pressure was 185; polymorphisms at this position were observed in 11.9% (5/42) of patients.

### 3.3. Phylogenetic Analysis

**Figure 2:**
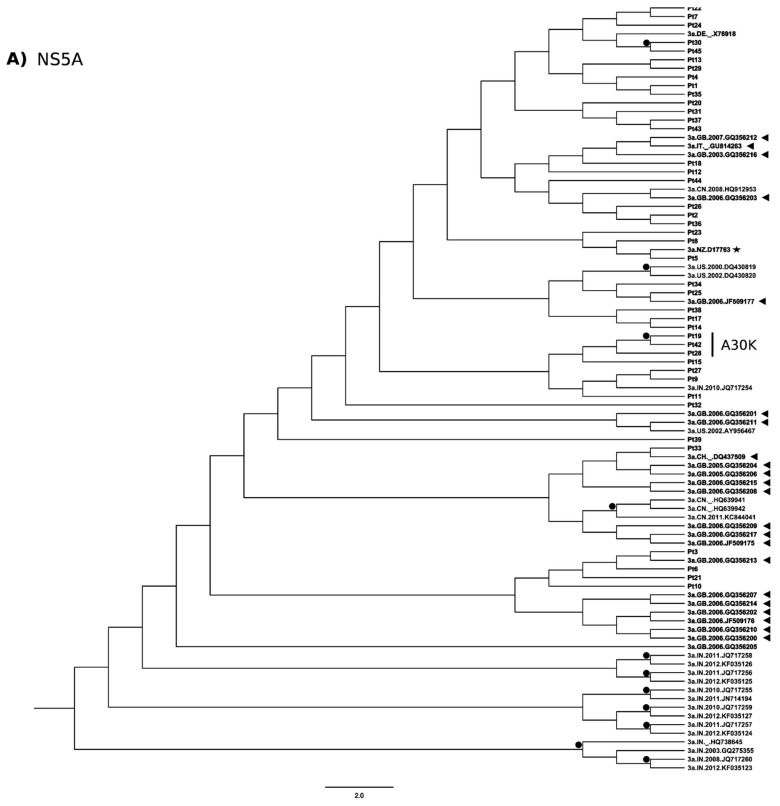

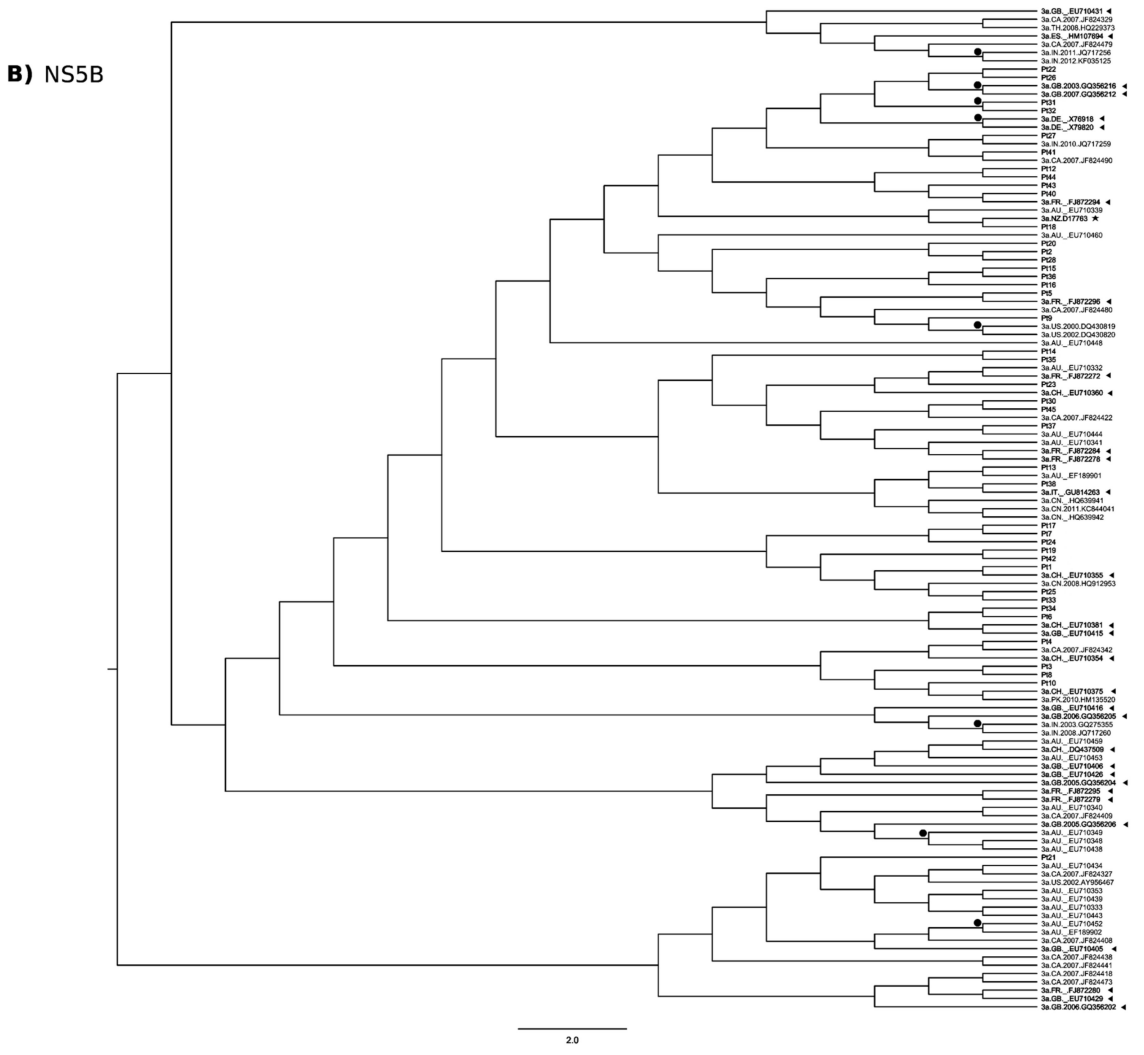
Phylogenetic trees of NS5A and NS5B. Phylogenetic analyses of: NS5A (**A**); and NS5B (**B**) sequences performed using 500 bootstrap repetitions. Only bootstrap values >70% are outlined with black circles. Sequences from the present study are indicated with patient (Pt) number; HCV sequences from European countries in the Los Alamos database are shown with black triangles. The country and year of these sequences are reported after the genotype. The GT3a reference sequence (D17763, in black star) is included. Patients harboring NS5A-A30K are side marked. AU, Australia; CA, Canada; CH, Switzerland; CN, China; DE, Germany; FR, France; GB, Great Britain; IN, India; ITA, Italy; PK, Pakistan; US, United States; NZ, New Zealand.

A total of 42 NS5A sequences derived from our Italian study patients and 47 GT3a sequences from North America, Europe and Asia were included in the phylogenetic analysis. Overall, several clusters of sequences appeared on the phylogenetic tree, showing apparent paraphyly, but there was no statistical support. Overall, sequences from Italian study patients were inter-dispersed with database sequences, with a tendency to group with sequences of European origin, although not supported by a significant bootstrap value (Figure 2A). Bootstrap significance was observed for sequences from two of the three patients harboring A30K (Pt19 and Pt42 but not Pt28) that were on the same branch of the phylogenetic tree.

For NS5B, Italian study patient sequences were less inter-dispersed between the database sequences (Figure 2B).

### 3.4. Analysis of Emergent Substitutions at Therapy Failure

Sanger sequencing results for the four patients who failed treatment are shown in Table 2. A more detailed analysis was performed using UDPS on samples from three patients experiencing treatment failure; no sufficient sample volume was available for Pt44. Overall, 33,684 reads were obtained (raw data). After data correction, the median read coverage per sample was 2056 (range 909–5437). All substitutions were tabulated and the number of reads for each amino acid position was specified (Table S2).

NS5A and NS5B nucleotide diversity in the three treatment failures was calculated (Table 4) and a progressive decrease of diversity was observed in all patients during failure history.

The dynamics of substitutions potentially involved in DAA resistance, as evidenced by the UDPS analysis, are shown in Table 4; UDPS results were concordant with qualitative results obtained by Sanger sequencing (98.3% for both NS5A and NS5B, considering all the amino acid positions in the sequenced genomic regions). Briefly, in Pt42, NS5A-L31F was detected in both failures (99.2% and 98.5%, respectively), while A30K, present at baseline at 92.0%, was replaced by A30T (not considered a DCV RAS [28]) at T1 with a frequency of 97.2%. This substitution was still detected after retreatment failure (T2, 98.4%). No RAS were detected in NS5B (Tables S2 and S3).

For Pt43, known RAS were detected in NS5A (Y93H, 99.2%) and NS5B (S282T, 95.8%) at T1. After re-treatment (T2), NS5A-Y93H was still the predominant substitution (99.5%), while NS5B-S282T was no longer detected. No additional RAS were observed. With respect to other NS5A positions potentially involved in DAA resistance, substitutions at positions 58 and 62 were also observed in nearly all viral quasispecies at the tested time points.

In Pt45, no substitutions were detected at positions known to reduce susceptibility to DAAs in both drug target regions.

When assessing substitutions not reported to be associated with drug resistance in Pt42, substitutions at NS5A positions 34 and 52 emerged at T1 and were also detected at T2 (Table 4). The NS5B substitution A306V was enriched at relapse; A306V represented 0.5% of the viral population at baseline and 95.3% at relapse (Table 4).

For Pt43, enrichment of NS5B-Q273P was observed: Q273P was detected at 3.0%, 99.8% and 98.9% at T0, T1 and T2, respectively.

In Pt45, the NS5B polymorphisms I184T, G188D and N310S represented minor viral populations at baseline (ca. 0.7%) and were enriched at T1 (>97%). These substitutions were linked on the same haplotype in various combinations (single, double or triple). The frequency of the triple substitution haplotype was below the UDPS threshold (0.2%) at baseline and 94.6% after virological failure.

## 4. Discussion

In this study, NS5A and NS5B genetic characteristics in 45 HCV GT3a Italian patients with advanced fibrosis or cirrhosis, who were treated with SOF-based therapies were analyzed. Twenty-three of these patients were treated with SOF + DCV ± RBV while 22 were treated with SOF + RBV ± IFN.

NS5A and NS5B polymorphisms were identified at amino acid positions not previously reported: three were in NS5A (T79S, T107K and T107S) and three were in NS5B (G166R, Q180K and C274W). However, none of these substitutions were observed in patients who failed DCV + SOF treatment.

The number of patients harboring known RAS at baseline was 11.9% for NS5A and 0% for NS5B. These findings are in agreement with data reported by Chen et al., where only 0.1% of sequences from all genotypes included in the Los Alamos database harbored RAS to SOF [30].

Among NS5A and NS5B positions associated with selective pressure in our study (Figure 1), T7 was one of the most polymorphic sites in NS5A, that was observed in 22 patients (52.4%), followed by E185 in NS5B, which was observed in five patients (11.9%).

Phylogenetic analysis revealed no distinct grouping of NS5A sequences. Only GT3 sequences harboring A30K tended to cluster together (Figure 2A), suggesting a possible common origin. The phylogenetic analysis further supported less genetic variability of NS5B.

Combination of DCV + SOF + RBV may overcome the presence of baseline NS5A RAS in cirrhotic patients, since two patients (Pt19, Pt28) achieved SVR despite harboring A30K, a substitution with reduced (44-fold over wild-type) susceptibility to DCV [31]. In contrast, Pt42 (Table 2 and Table 4) with baseline NS5A-A30K relapsed after treatment with DCV + SOF; however, A30K was replaced by A30T at failure, while L31F emerged de novo. In vitro susceptibility data indicate that A30T does not confer DCV resistance, while L31F alone and combined with A30T confers high level DCV resistance (585- and 675-fold, respectively, vs. wild-type) [28].

Baseline polymorphisms at position 58 (P58A/S/T) were detected in three patients, and only NS5A-P58S was observed in Pt43 who subsequently failed treatment with DCV + SOF (Table 2 and Table 4). However, a recent study [28] declared that P58S did not have any impact on DCV activity in GT3a-infected patients, while it is reported to confer resistance to NS5A inhibitors in GT6-infected patients [32].

NS5A-A62 substitutions (A62P/S/T) did not seem to impact therapy response, since seven patients with these polymorphisms achieved SVR.

NS5A-Y93 substitutions (Y93D/N) were observed in one patient (Pt28) who also harbored A30K. This patient was cirrhotic, received SOF + DCV + RBV, and achieved SVR. The novel Y93D substitution, detected in 2 of 42 patients, has not been reported previously, and although its DCV susceptibility is unknown, it did not seem to impact treatment response.

The longitudinal UDPS analysis of patients who did not achieve SVR (Pt42, Pt43 and Pt45) highlighted the dynamics of viral quasispecies. The diversity was lower after failure as expected (Table 3), but the reduction was not significant, presumably due to the low number of analyzed patients.

In one patient with virological failure (Pt42), NS5A-L31F emerged; this substitution has only been reported to emerge under DCV selection in vitro [33]. In our GT3 patient, L31F was linked to emergent A30T (Table 4  and Table S2), perhaps suggestive of an adaptive role in DCV resistance [28,34].

No minor substitutions were observed in addition to NS5A-A62P/S/T (Table 4 and Table S2). Y93C and Y93F were observed at baseline with a frequency of 1.8% and 1.1% respectively (Table 4), but both of these polymorphisms were not detected at subsequent time points.

The signature SOF RAS NS5B-S282T was observed in Pt43 at first virological failure to SOF + DCV, but was not detected (by Sanger sequencing or UDPS) at baseline or at failure to re-treatment with SOF + RBV (Table 2 and Table S2).

Further studies are required to address possible significance of the combination of NS5A and NS5B substitutions observed at failure to SOF + DCV treatment, as well as their fitness and possible selective advantage. In addition, possible linkage of NS5A and NS5B to substitutions within distant genomic regions, such as NS3, may play a role in the future, considering the upcoming GT3 therapeutic regiments, including new protease inhibitors.

Overall, high concordance between Sanger sequencing and UDPS was observed; however, UDPS allowed investigating the presence of minor substitutions, and their frequency changes after therapy failure. In fact, some NS5B polymorphisms, like G188D, Q273P, L285F, A306V, N310S, not previously reported in the public databases of HCV sequences, represented minor substitutions at baseline (<1%), that were enriched after virological failure. However, their significance to SOF failure is unclear and would need further investigation. In Pt45, who failed treatment without known RAS, three baseline polymorphisms, present at low-frequency (I184T, G188D and N310S, each detected at ca. 0.7%), were observed on the same haplotype, and became the predominant species at virological failure. Studies on GT3 replicon harboring NS5B I184T-G188D-N310S showed that the SOF activity was similar against these substitutions as wild-type (data not shown).

The main limitation of this study is the small number of virological failures analyzed. Furthermore, analysis of minor viral populations at baseline in patients achieving SVR was not performed. Overall, a full understanding of the role of some of these minor substitutions may be hard to determine, given the high rate of virological success of SOF-based regimens and the rare occurrence of RAS emergence in patients not achieving SVR.

## Figures and Tables

**Table 1:** Baseline demographics and disease characteristics of the study population.

		Total	SOF + DCV	SOF + DCV + RBV	SOF + RBV	SOF + P-R	Not Known
**Number of patients (F/M)**		45	7	16	12	9	1
		(7/38)	(0/7)	(1/15)	(2/10)	(4/5)	(0/1)
**Median Age, years** **(range)**		56	56	56	57	56	n.a.
		(42–69)	(49–63)	(42–63)	(52–69)	(47–66)	
**HCV RNA (Log_10_ IU/mL)** **Median (Min–Max)**		5.3	5.0	5.2	5.5	5.9	5.4
		(1.1–7.0)	(1.1–7.0)	(4.0–6.8)	(3.8–6.7)	(3.2–6.7)	
**HIV positive patients** **(F/M)**		3	0	1	2	0	0
		(0/3)	(0/0)	(0/1)	(0/2)	(0/0)	(0/0)
Number of patients without cirrhosis * (F/M)		9	2	3	3	1	n.a.
		(0/9)	(0/2)	(0/3)	(0/3)	(0/1)	
Number of patients with cirrhosis * (F/M)		35	5	13	9	8	n.a.
		(7/28)	(0/5)	(1/12)	(2/7)	(4/4)	
**Prior pegIFN experience**	**Treatment–naive** **(F/M)**	26	5	8	6	6	1
		(3/23)	(0/5)	(0/8)	(1/5)	(2/4)	(0/1)
	**Treatment–experienced** **(F/M)**	19	2	8	6	3	0
		(4/15)	(0/2)	(1/7)	(1/5)	(2/1)	(0/0)

HCV = hepatitis C virus; HIV = human immunodeficiency virus; SOF = sofosbuvir; DCV = daclatasvir; RBV = ribavirin; P-R = pegylated interferon (pegIFN) + ribavirin (RBV); n.a. = not available; F = female; M = male; HIV = human immunodeficiency virus. * Based on clinical or histological features or with non-invasive assessment by transient elastography (stiffness > 14 KPa).

**Table 2:** Characteristics of HCV genotype (GT)3a patients who failed DAA treatment and their drug resistance-associated substitutions detected by Sanger sequencing.

Pt	Fibrosis Stage	Prior IFN Treatment Experience (Response)	DAA	Baseline HCV RNA (LogIU/mL)	Antiviral Regimen (wks)	Response	Known RAS Detected at Failure by Sanger Sequencing	
							NS5A	NS5B
**42**	3	Naïve	**First DAA regimen**	7.0	SOF + DCV (12)	Relapser	L31F	none
			**Second DAA regimen**	7.0	SOF + DCV + RBV (24)	Relapser	L31F	none
**43**	4	Naïve	**First DAA regimen**	5.0	SOF + DCV (24)	Relapser	P58S Y93H	S282T
			**Second DAA regimen**	5.8	SOF + RBV (48)	Relapser	P58S Y93H	none
**44**	4	P-R (Null responder)	**First DAA regimen**	5.9	SOF + RBV (24)	Relapser	none	none
			**Second DAA regimen**	6.3	SOF + DCV + RBV (24)	SVR12	n.a.	n.a.
**45**	4	P-R (Null responder)	**First DAA regimen**	4.6	SOF + RBV (24)	Relapser	none	none
			**Second DAA regimen**	4.1	SOF + DCV + RBV (24)	SVR12	n.a.	n.a.

Pt = patient; DAA = direct antiviral treatment; wks = weeks; RAS = resistance-associated substitutions.

**Table 3:** Amino acid (aa) substitutions detected at baseline by Sanger sequencing in non-structural protein 5A (NS5A) and non-structural protein 5B (NS5B) from 42 patients for whom sequence data are available. Substitutions that differed from the reference sequence (D17763) are reported. The numbers in subscript text next to the amino acid substitution represent the frequency of each polymorphism. Substitutions known to reduce susceptibility to DAAs in HCV GT3a patients are shown in bold.

**Non-Structural Protein 5A (NS5A)**																
**Reference**	D_40.5_	D_92.9_	R_95.2_	T_52.4_	D_97.6_	S_83.3_	L_100.0_	A_4.8_	K_97.6_	A_9.5_	L_97.6_	K_97.6_	M_97.6_	**A_90.5_**	L_97.6_	I_97.6_
**aa**	2	3	6	7	10	14	16	17	20	21	23	26	28	**30**	34	37
**Variants _%_**	G_61.9_	E_7.1_	H_4.8_	A_2.4_	E_2.4_	I_2.4_	V_2.4_	S_90.5_	R_2.4_	T_92.9_	I_2.4_	R_2.4_	L_2.4_	**K_9.5_**	I_2.4_	L_2.4_
		N_2.4_		D_23.8_		L_2.4_		T_2.4_								
				I_14.3_		T_16.7_		Y_2.4_								
				V_16.7_												
**Reference**	K_95.2_	Y_97.6_	K_97.6_	V_97.6_	M_100.0_	S_97.6_	T_97.6_	P_92.9_	A_2.4_	I_97.6_	T_88.1_	V_100.0_	L_97.6_	A_92.9_	P_97.6_	T_90.5_
**aa**	41	43	44	46	53	54	55	58	62	63	64	67	74	75	77	79
**Variants _%_**	R_4.8_	F_2.4_	R_2.4_	A_2.4_	V_2.4_	A_2.4_	A_2.4_	A_2.4_	P_2.4_	L_2.4_	A_7.1_	I_2.4_	I_2.4_	V_9.5_	S_2.4_	A_2.4_
				L_2.4_				S_2.4_	S_85.7_		S_4.8_	M_2.4_				M_2.4_
								T_2.4_	T_14.3_							R_2.4_
																S_2.4_
**Reference**	M_97.6_	H_73.8_	**Y_95.2_**	S_66.7_	S_54.8_	P_97.6_	T_83.3_	W_97.6_	N_90.5_	S_97.6_						
**aa**	83	85	**93**	98	103	104	107	111	116	117						
**Variants _%_**	T_4.8_	C_2.4_	D_4.8_	G_35.7_	A_4.8_	L_2.4_	A_2.4_	L_2.4_	S_9.5_	G_2.4_						
		R_2.4_	**H_2.4_**		P_45.2_		K_2.4_									
		Y_28.6_	N_2.4_				N_2.4_									
							S_7.1_									
**Non-Structural Protein 5B (NS5B)**																
**Reference**	P_95.2_	G_100.0_	V_100_	Q_97.6_	I_90.5_	E_88.1_	T_95.2_	G_97.6_	P_31.0_	K_81.0_	S_95.2_	T_92.9_	Q_97.6_	V_95.2_	I_97.6_	N_90.5_
**aa**	156	166	169	180	184	185	186	188	189	206	210	213	231	235	239	244
**Variants _%_**	A_4.8_	R_2.4_	I_2.4_	K_2.4_	L_4.8_	A_11.9_	A_2.4_	S_2.4_	A_4.8_	E_2.4_	A_4.8_	A_4.8_	R_2.4_	G_2.4_	V_2.4_	D_11.9_
					T_4.8_	G_2.4_	V_2.4_		S_64.3_	Q_14.3_		N_2.4_		M_4.8_		
										T_2.4_						
**Reference**	E_97.6_	R_90.5_	S_97.6_	S_97.6_	F_97.6_	K_97.6_	A_85.7_	Q_95.2_	C_100.0_	I_95.2_	T_100.0_	K_76.2_	N_0.0_	R_97.6_	N_97.6_	F_97.6_
**aa**	246	250	254	255	267	270	272	273	274	293	300	304	307	309	310	313
**Variants _%_**	Q_2.4_	K_11.9_	T_2.4_	A_2.4_	Y_2.4_	R_2.4_	D_16.7_	P_4.8_	W_2.4_	L_4.8_	S_2.4_	R_26.2_	G_97.6_	Q_2.4_	D_4.8_	L_2.4_
													S_2.4_			

**Table 4:** Amino acid substitutions observed in NS5A and NS5B by ultra-deep pyrosequencing (UDPS) in patients who failed treatment. Only substitutions at positions involved in DAA resistance and/or showing relevant changes in frequency are reported. A threshold of 1% was applied. Only for those substitution that showed enrichment at virological failure lower baseline BL frequencies (i.e., 0.7%) were considered.

**Non-Structural Protein 5A (NS5A)**						
	**Ref.**	**Amino-Acid Position**	**Variant**	**Baseline n° Reads (Frequency, %)**	**T1 n° Reads (Frequency, %)**	**T2 n° Reads (Frequency, %)**
**Pt42**	A	30	K	847 (92.0)	n.d.	n.d.
			R	72 (7.8)	12 (1.3)	n.d.
			T	n.d.	884 (97.2)	1526 (98.4)
	L	31	F	n.d.	902 (99.2)	1527 (98.5)
	L	34	I	n.d.	898 (98.8)	1536 (99.1)
	V	52	M	n.d.	585 (64.4)	1530 (98.741)
	A	62	S	915 (99.3)	894 (98.3)	1539 (99.3)
	T	87	P	n.d.	672 (74.0)	n.d.
	Y	93	F	10 (1.1)	n.d.	n.d.
	**Diversity** (nt substitutions per site ×10^−4^)			173.6	112.8	80.8
**Pt43**	P	58	M	n.d.	n.d.	29 (1.26)
			S	2289 (99.1)	2966 (99.2)	5401 (99.4)
	A	62	S*	2300 (99.6)	2983 (99.7)	5421 (99.7)
	Y	93	C	42 (1.8)	n.d.	n.d.
			H	n.d.	2968 (99.2)	5408 (99.5)
	**Diversity** (nt substitutions per site ×10−4)			373.4	53.8	59.0
**Non-Structural Protein 5B (NS5B)**						
	**Ref.**	**Amino-Acid Position**	**Variant**	**Baseline n° Reads Frequency, %)**	**T1 n° Reads (Frequency, %)**	**T2 n° Reads (Frequency, %)**
**Pt42**	G	188	A	946 (45.1)	n.a.	n.d.
	K	212	R	944 (45.0)	n.a.	n.d.
	N	244	D	364 (17.4)	n.a.	n.d.
	K	304	R	540 (51.1)	n.a.	n.d.
	A	306	V	5 (0.5)	n.a.	888 (95.3)
	**Diversity** (nt substitutions per site ×10^−4^)			276.4	n.a.	62.7
**Pt43**	I	160	V	n.d.	4 (0.4)	1120 (94.0)
	Q	273	P	62 (3.0)	1971 (99.7)	2670 (99.0)
	S	282	T	n.d.	1892 (95.7)	n.d.
	L	285	F	n.d.	1920 (97.2)	2677 (99.2)
	**Diversity** (nt substitutions per site ×10^−4^)			232.2	59.6	53.1
**Pt45**	L	159	P	n.d.	5 (0.54)	n.a.
	I	184	T	15 (0.7)	2001 (97.32)	n.a.
			V	1312 (60.3)	n.d.	n.a.
	G	188	D	16 (0.7)	1995 (97.03)	n.a.
	P	189	S	2169 (99.6)	2049 (99.66)	n.a.
	K	206	E	1311 (60.2)	n.d.	n.a.
	V	235	M	693 (31.8)	n.d.	n.a.
	N	307	G	1221 (99.9)	1134 (99.6)	n.a.
	N	310	S	8 (0.6)	1111 (97.6)	n.a.
	**Diversity** (nt substitutions per site ×10^−4^)			384.0	96.4	n.a.

* S is present in the Food and Drug Administration (FDA) reference sequence (GU814263). Ref. = reference; T1 = first treatment failure; T2 = second treatment failure; Pt = patient; n.a. = not applicable; n.d. = not determined.

## Supplementary Materials

The following are available online at www.mdpi.com/1999-4915/9/8/212/s1.
