# Supplementary material for: Characterization of Naturally Occurring NS5A and NS5B Polymorphisms in Patients Infected with HCV Genotype 3a Treated with Direct-Acting Antiviral Agents
Source: Viruses. 2017 Aug 7;9(8):212. doi: 10.3390/v9080212 (PMC5580469; doi:10.3390/v9080212)
Supplement: Supplementary file 1 [file viruses-09-00212-s001.pdf]

SUPPLEMENTARY MATERIAL

Supplementary Table S1: Prevalence of polymorphisms observed in the NS5A (panel A) and NS5B (panel B) regions from 53 and 233 sequences, respectively, retrieved from the Los Alamos database. The reference D17763 is shown on the top line. The numbers next to the observed amino-acid substitutions represent the percent of observations with a specific substitution.

A)

**Reference**	$S_{96.2}$	$D_{13.2}$	$D_{98.1}$	$T_{83.0}$	$D_{92.5}$	$C_{98.1}$	$S_{94.3}$	$V_{98.1}$	$L_{98.1}$	$A_{7.5}$	$F_{98.1}$	$K_{94.3}$	$A_{98.1}$	$I_{98.1}$	$M_{98.1}$	$A_{98.1}$	$I_{92.5}$
**aa**	1	2	3	7	10	13	14	15	16	17	19	20	21	27	28	30	37
**Variants** %	$C_{3.8}$	$G_{86.8}$	$N_{1.9}$	$D_{17.0}$	$E_{7.5}$	$S_{1.9}$	$I_{5.7}$	$A_{1.9}$	$V_{1.9}$	$S_{92.5}$	$S_{1.9}$	$R_{5.7}$	$S_{1.9}$	$V_{1.9}$	$I_{1.9}$	$L_{1.9}$	$L_{7.5}$
				$I_{9.4}$			$M_{34.0}$	$L_{3.8}$					$T_{92.5}$			$T_{3.8}$	$V_{1.9}$
				$P_{1.9}$			$T_{37.7}$	$M_{1.9}$								$V_{1.9}$	
				$S_{1.9}$				$T_{9.4}$									

**Reference**	$K_{92.5}$	$Y_{98.1}$	$V_{98.1}$	$M_{98.1}$	$S_{98.1}$	$P_{98.1}$	$A_{94.3}$	$A_{98.1}$	$I_{98.1}$	$T_{69.8}$	$S_{98.1}$	$L_{98.1}$	$A_{96.2}$	$T_{96.2}$	$W_{98.1}$	$H_{96.2}$	$S_{84.9}$
**aa**	41	43	46	53	54	58	61	62	63	64	71	74	75	79	84	85	98
**Variants** %	$R_{7.5}$	$S_{1.9}$	$A_{1.9}$	$T_{1.9}$	$T_{1.9}$	$R_{1.9}$	$S_{5.7}$	$P_{1.9}$	$L_{1.9}$	$A_{30.2}$	$T_{1.9}$	$I_{1.9}$	$S_{3.8}$	$K_{3.8}$	$C_{1.9}$	$N_{3.8}$	$G_{15.1}$
						$S_{1.9}$		$Q_{1.9}$		$S_{3.8}$				$M_{5.7}$		$Y_{32.1}$	
								$S_{60.4}$						$R_{1.9}$			
								$T_{32.1}$									

**Reference**	$T_{98.1}$	$S_{92.5}$	$P_{96.2}$	$Y_{98.1}$	$T_{98.1}$	$W_{92.5}$	$N_{96.2}$	$S_{98.1}$	$Y_{98.1}$	$V_{98.1}$
**aa**	99	103	104	106	107	111	116	117	118	119
**Variants** %	$A_{1.9}$	$A_{7.5}$	$H_{3.8}$	$F_{1.9}$	$A_{1.9}$	$L_{7.5}$	$D_{3.8}$	$N_{1.9}$	$C_{1.9}$	$A_{1.9}$
	$I_{3.8}$	$P_{45.3}$	$L_{1.9}$				$S_{11.3}$			

B)

**Reference**	**P**$_{97.0}$	**A**$_{99.1}$	**Y**$_{99.6}$	**D**$_{99.6}$	**V**$_{98.7}$	**R**$_{99.6}$	**A**$_{98.7}$	**Y**$_{99.1}$	**D**$_{99.1}$	**V**$_{98.7}$	**I**$_{98.3}$	**Q**$_{99.1}$	**K**$_{96.1}$	**L**$_{99.1}$	**S**$_{98.3}$	**I**$_{89.3}$
**aa**	156	157	162	164	169	173	174	176	177	178	179	180	181	182	183	184
**Variants** %	A$_{3.4}$	P$_{0.4}$	F$_{0.4}$	G$_{0.4}$	I$_{1.3}$	M$_{0.4}$	V$_{1.3}$	D$_{0.8}$	K$_{0.8}$	A$_{0.8}$	A$_{0.4}$	E$_{0.8}$	Q$_{2.1}$	M$_{0.8}$	A$_{0.8}$	K$_{0.4}$
	R$_{0.4}$	S$_{0.4}$								I$_{0.4}$	L$_{0.4}$		R$_{1.7}$		P$_{0.8}$	S$_{0.4}$
	S$_{0.4}$										R$_{0.8}$					T$_{0.8}$
																V$_{1.7}$

**Reference**	**E**$_{69.5}$	**T**$_{96.1}$	**M**$_{98.7}$	**G**$_{99.6}$	**P**$_{35.6}$	**A**$_{99.1}$	**Q**$_{98.7}$	**Q**$_{99.6}$	**E**$_{96.6}$	**R**$_{96.1}$	**L**$_{99.6}$	**K**$_{76.4}$	**M**$_{98.7}$	**T**$_{98.3}$	**S**$_{97.9}$	**K**$_{99.6}$
**aa**	185	186	187	188	189	190	198	199	202	203	204	206	207	209	210	211
**Variants** %	A$_{28.3}$	A$_{2.1}$	I$_{0.4}$	V$_{0.4}$	A$_{2.1}$	S$_{0.8}$	A$_{0.4}$	E$_{0.4}$	D$_{3.4}$	F$_{0.4}$	M$_{0.4}$	D$_{0.4}$	A$_{0.4}$	A$_{0.4}$	A$_{2.1}$	R$_{0.4}$
	G$_{1.3}$	I$_{0.4}$	L$_{0.8}$		D$_{0.8}$		K$_{0.4}$			H$_{2.5}$		E$_{15.5}$	I$_{0.8}$	G$_{0.4}$		
	T$_{0.4}$	S$_{0.4}$			L$_{1.3}$		R$_{0.4}$			Y$_{0.8}$		N$_{1.3}$		S$_{0.8}$		
		V$_{0.8}$			S$_{61.6}$							Q$_{6.9}$				
					T$_{0.8}$							R$_{0.4}$				
												T$_{3.0}$				

**Reference**	**K**$_{96.6}$	**T**$_{94.4}$	**L**$_{97.9}$	**S**$_{99.6}$	**D**$_{99.6}$	**D**$_{99.6}$	**V**$_{99.1}$	**E**$_{99.6}$	**Q**$_{99.1}$	**I**$_{97.4}$	**V**$_{93.1}$	**E**$_{99.1}$	**E**$_{97.4}$	**I**$_{97.4}$	**C**$_{99.6}$	**N**$_{86.3}$
**aa**	212	213	215	218	220	225	228	230	231	233	235	237	238	239	242	244
**Variants** %	E$_{0.4}$	A$_{1.3}$	M$_{2.1}$	L$_{0.4}$	G$_{0.4}$	Y$_{0.4}$	I$_{0.8}$	G$_{0.4}$	H$_{0.4}$	F$_{0.8}$	A$_{2.1}$	G$_{0.8}$	A$_{0.4}$	M$_{0.4}$	A$_{0.4}$	A$_{0.8}$
	R$_{3.0}$	D$_{0.4}$							R$_{0.4}$	T$_{0.4}$	M$_{1.7}$		D$_{1.7}$	V$_{2.1}$		D$_{12.0}$
		I$_{0.8}$								V$_{1.3}$	T$_{3.0}$		S$_{0.4}$			H$_{0.4}$
		N$_{1.3}$														K$_{0.4}$
		S$_{1.3}$														S$_{0.8}$
		V$_{0.4}$														T$_{0.4}$

**Reference**	**L**$_{99.6}$	**E**$_{99.1}$	**P**$_{99.6}$	**A**$_{99.1}$	**R**$_{86.3}$	**K**$_{97.9}$	**V**$_{99.1}$	**I**$_{99.6}$	**S**$_{95.7}$	**S**$_{99.6}$	**E**$_{99.6}$	**C**$_{99.1}$	**M**$_{99.1}$	**F**$_{97.4}$	**S**$_{98.7}$	**K**$_{99.1}$
**aa**	245	246	247	249	250	251	252	253	254	255	258	262	266	267	269	270
**Variants** %	P$_{0.4}$	D$_{0.4}$	Q$_{0.4}$	P$_{0.4}$	G$_{0.4}$	Q$_{0.4}$	A$_{0.8}$	V$_{0.4}$	C$_{0.4}$	A$_{0.4}$	D$_{0.4}$	I$_{0.4}$	R$_{0.8}$	H$_{0.8}$	G$_{0.4}$	R$_{0.8}$

		P$_{0.4}$		V$_{0.4}$	K$_{13.3}$	R$_{1.3}$			H$_{0.4}$			V$_{0.4}$		N$_{0.4}$	N$_{0.8}$	T$_{0.4}$
						T$_{0.4}$			T$_{3.4}$					T$_{0.4}$		
														Y$_{0.8}$		

**Reference**	**A**$_{91.8}$	**Q**$_{93.6}$	**C**$_{100.0}$	**Y**$_{99.6}$	**R**$_{99.6}$	**S**$_{98.7}$	**V**$_{99.6}$	**L**$_{99.1}$	**P**$_{99.6}$	**S**$_{98.7}$	**F**$_{99.6}$	**I**$_{97.0}$	**Y**$_{99.1}$	**I**$_{99.6}$	**T**$_{96.1}$	**A**$_{97.4}$
**aa**	272	273	274	276	277	282	284	285	286	288	289	293	296	297	300	303
**Variants** %	D$_{3.4}$	H$_{1.3}$	R$_{0.4}$	D$_{0.4}$	L$_{0.4}$	N$_{0.4}$	I$_{0.4}$	F$_{0.8}$	T$_{0.4}$	H$_{0.4}$	M$_{0.4}$	L$_{0.8}$	F$_{0.4}$	T$_{0.4}$	L$_{0.4}$	C$_{0.4}$
	I$_{0.8}$	P$_{3.4}$				R$_{0.8}$				N$_{0.4}$		M$_{1.3}$	H$_{0.4}$		S$_{3.4}$	S$_{0.4}$
	L$_{1.3}$	R$_{0.4}$								R$_{0.4}$		V$_{0.8}$				V$_{1.7}$
	Q$_{0.4}$	S$_{1.3}$														
	T$_{1.3}$															
	V$_{0.8}$															

**Reference**	**K**$_{39.9}$	**A**$_{94.8}$	**A**$_{98.7}$	**N**$_{0.0}$	**L**$_{66.9}$	**R**$_{95.3}$	**N**$_{93.6}$	**P**$_{98.7}$	**D**$_{98.3}$	**F**$_{99.6}$	**V**$_{99.6}$	**L**$_{99.6}$	**V**$_{99.6}$
**aa**	304	305	306	307	308	309	310	311	312	313	315	320	322
**Variants** %	N$_{1.3}$	G$_{0.4}$	S$_{1.3}$	G$_{100.0}$	I$_{0.4}$	K$_{0.8}$	D$_{3.0}$	F$_{0.8}$	E$_{0.8}$	M$_{0.4}$	I$_{0.4}$	Q$_{0.4}$	L$_{0.4}$
	R$_{60.1}$	S$_{0.4}$				Q$_{3.4}$	H$_{0.8}$	S$_{0.4}$	S$_{0.4}$				
		V$_{4.2}$				V$_{0.4}$	S$_{2.5}$		V$_{0.4}$				

Supplementary Table S2: Type and frequency of amino-acid substitutions detected by UDPS in NS5A (panel A) and NS5B (panel B) at baseline and treatment failure for three patients. Substitutions that differed from the reference D17763 (top sequence), are shown while the frequency of the substitution in the viral population and the number of reads harboring the substitution/total number of reads is shown in parenthesis. Only substitutions showing enrichment at treatment failure are included and frequencies lower than the threshold of 1% are reported.

A)
Pt42

aa ref	Position	T0		T1		T2	
D	2	G 99.6% (917/921)		G 99.0% (900/909)		G 99.0% (1534/1550)	
D	3			G 2.1% (19/909)			
T	7	D 98.6% (908/921)		D 98.7% (897/909)		D 97.9% (1518/1550)	
I	8			V 1.4% (13/909)			
W	11			S 5.1% (46/909)			
A	17	F 5.1% (47/921)	S 94.6% (871/921)	S 99.2% (902/909)		S 98.8% (1532/1550)	
K	26	R 2.9% (27/921)					
A	30	K 92.0% (847/921)	R 7.8% (72/921)	R 1.3% (12/909)	T 97.2% (884/909)	T 98.4% (1526/1550)	
L	31			F 99.2% (902/909)		F 98.5% (1527/1550)	
L	34			I 98.8% (898/909)		I 99.1% (1536/1550)	
G	42					V 4.6% (71/1550)	
V	46	L 1.3% (12/921)					
W	47	C 1.8% (17/921)		C 3.5% (32/909)		C 2.8% (43/1550)	
R	48	P 4.7% (43/921)		P 5.7% (52/909)			
D	50	Y 54.7% (504/921)		Y 35.6% (324/909)		Y 25.9% (401/1550)	
V	52			M 64.4% (585/909)		M 98.7% (1530/1550)	
G	60	R 1.2% (11/921)					
A	61			V 1.1% (10/909)			
A	62	S 99.4% (915/921)		S 98.4% (894/909)		S 99.3% (1539/1550)	
K	68					E 1.0% (16/1550)	
N	69			D 1.5% (14/909)			
M	72	V 1.1% (10/921)		I 1.1% (10/909)			

R	78	C 1.3% (12/921)		C 1.1% (10/909)			
H	85	L 1.2% (11/921)					
T	87			P 73.9% (672/909)			
P	89					S 1.6% (24/1550)	
I	90			V 2.8% (25/909)			
Y	93	F 1.1% (10/921)					
S	98	G 4.0% (8/199)		G 100.0% (182/182)		G 99.8% (607/608)	
P	100	H 1.0% (2/199)					
P	104	S 6.0% (12/199)					

pt43

aa ref	Position	T0		T1		T2	
D	3	N 99.4% (2294/2309)		N 97.2% (2906/2991)		N 98.8% (5370/5436)	
R	6	H 99.5% (2297/2309)		H 96.0% (2873/2991)		H 97.7% (5313/5436)	
T	7	V 57.6% (1330/2309)		V 98.3% (2939/2991)		V 99.0% (5384/5436)	
D	10					R 2.4% (55/2309)	
W	11	S 7.1% (163/2309)		S 16.6% (495/2991)			
S	14	P 3.5% (81/2309)	R 2.4% (55/2309)				
A	17	S 99.5% (2297/2309)		S 99.5% (2976/2991)		S 99.0% (5379/5436)	
A	21	T 98.4% (2272/2309)		T 99.2% (2968/2991)		T 99.1% (5389/5436)	
I	27					V 98.8% (5370/5436)	
W	47	C 9.2% (213/2309)		C 3.5% (106/2991)		C 14.2% (774/5436)	
T	55			A 1.2% (37/2991)		A 1.0% (55/5436)	
P	58	S 99.1% (2289/2309)		S 99.2% (2966/2991)		S 99.4% (5401/5436)	M 1.3% (29/2309)
A	62	S 99.6% (2300/2309)		S 99.7% (2983/2991)		S 99.7% (5421/5436)	

V	67	I 19.1% (442/2309)	M 1.3% (29/2309)				
A	75	V 56.7% (1310/2309)		V 99.6% (2978/2991)		V 99.6% (5417/5436)	
Y	93	C 1.8% (42/2309)		H 99.2% (2968/2991)		H 99.5% (5408/5436)	
S	98	G 100.0% (1019/1019)		G 99.9% (1348/1349)		G 99.9% (2286/2289)	
S	103	P 99.6% (1015/1019)		P 99.8% (1347/1349)		P 99.8% (2284/2289)	
T	107	A 5.0% (51/1019)					

B)
Pt42

aa ref	Position	T0		T1		T2	
K	181	E 1.2% (25/2096)					
G	188	A 45.1% (946/2096)					
P	189	S 99.2% (2079/2096)				S 99.3% (1732/1745)	
K	206	Q 2.4% (50/2096)					
K	211					R 1.8% (31/1745)	
K	212	R 45.0% (944/2096)					
T	213	N 1.5% (31/2096)					
N	244	D 17.4% (364/2096)					
G	264	V 1.0% (22/2096)				V 22.6% (394/1745)	
A	272					V 1.5% (26/1745)	
K	304	R 51.1% (540/1057)					
A	306	V 0.5% (5/1057)				V 95.3% (888/932)	
N	307	G 99.8% (1055/1057)				G 99.6% (928/932)	

pt43

aa ref	Position	T0		T1		T2	
I	160			V 0.4% (4/942)		V 94.0% (1120/1192)	
P	189	S 99.5% (2038/2048)		S 99.4%		S 99.7% (2690/2699)	

				(1964/1976)			
E	202			D 2.6% (51/1976)			
M	207	V 2.2% (46/2048)					
T	209					A 2.5% (67/2699)	
K	211	R 2.6% (53/2048)		R 2.2% (43/1976)	S 1.2% (23/1976)		
T	213	N 2.2% (45/2048)	P 1.8% (36/2048)			P 1.7% (47/2699)	
L	215	S 6.2% (127/2048)	V 1.0% (21/2048)	S 1.2% (24/1976)		S 3.0% (80/2699)	
V	228			A 1.1% (22/1976)			
V	235	M 9.9% (202/2048)					
E	237					G 1.8% (50/2699)	
L	260	P 1.1% (22/2048)		R 1.1% (21/1976)			
G	263			R 1.1% (22/1976)			
G	264	V 1.4% (28/2048)		V 1.8% (35/1976)			
K	270					R 1.2% (33/2699)	
A	272			V 3.3 (66/1976)			
Q	273	P 3.0% (62/2048)		P 99.8% (1971/1976)		P98.9% (2670/2699)	
S	282			T95.8% (1892/1976)			
L	285			F 97.2% (1920/1976)		F 99.2% (2677/2699)	
P	286					S 6.6% (177/2699)	
S	288	N 5.1% (104/2048)		C 2.1% (42/1976)		C 1.1% (30/2699)	
K	298	R 1.5% (30/2048)					
K	304	R 1.9% (23/1189)					
N	307	G 99.9% (1188/1189)		G 99.5% (1029/1034)		G 98.2% (1480/1507)	S 1.8% (27/1507)

pt45

aa ref	Position	T0		T1		T2	
I	184	T 0.7% (15/2177)	V 60.27% (1312/2177)	T 97.3% (2001/2056)			
M	187	T 1.2% (25/2177)					

G	188	D 0.7% (16/2177)		D 97.0% (1995/2056)			
P	189	S 99.6% (2169/2177)		S 99.7% (2049/2056)			
K	206	E 60.2% (1311/2177)					
K	211			N 2.3% (47/2056)			
K	212	E 2.0% (43/2177)		E 1.4% (28/2056)			
T	213	I 22.1% (481/2177)					
L	215	S 2.7% (59/2177)					
F	217			V 15.8% (325/2056)			
V	235	M 31.8% (693/2177)					
G	271	R 2.7% (59/2177)					
A	272	G 36.7% (798/2177)		D 39.0% (802/2056)			
Q	273	P 73.9% (1609/2177)		P 45.8% (942/2056)			
K	304	R 22.3% (273/1222)					
N	307	G 99.9% (1221/1222)		G 99.6% (1134/1138)			
N	310	S 0.6% (8/1222)		S 97.6% (1111/1138)			

Aa=amino acid; ref=reference; T0=baseline; T1=the first treatment failure visit; T2=the second treatment failure visit.

Supplementary Table S3. Baseline characteristics for all HCV GT3a study patients.

Pt	LT	Fibrosis state *	HIV status	HCV RNA Log IU/mL	Prior P-R treatment experience	DAA-based Treatment (wks)	Therapy response **	Polymorphisms at position potentially involved in DAA resistance	
								NS5A	NS5B
1	no	4	neg	4.04	Naive	SOF+RBV (24)	SVR24	None	N244D
2	no	4	neg	6.04	Experienced	SOF+DCV+RBV (24)	SVR12	A62T	None
3	no	4	neg	4.77	Experienced	SOF+pIFN+RBV (12)	SVR24	Y93D	None
4	no	4	neg	6.35	Experienced	SOF+pIFN+RBV (12)	SVR24	None	None
5	no	4	neg	5.97	Naive	SOF+pIFN+RBV (24)	SVR24	A62T	None
6	no	4	neg	4.76	Naive	SOF+pIFN+RBV (12)	SVR24	P58T A62T	None
7	no	4	neg	6 .74	Naive	SOF+pIFN+RBV (12)	SVR24	None	None
8	no	4	neg	4 .16	Naive	SOF+pIFN+RBV (12)	SVR24	None	R309Q
9	yes	4	neg	4 .76	Naive	SOF+DCV (20)	SVR24	None	None
10	yes	1	neg	6 .37	Naive	SOF+pIFN+RBV (24)	SVR24	P58A A62T Y93H	None
11	no	4	neg	6 .03	Experienced	SOF+DCV (24)	SVR12	None	n.d.
12	yes	4	neg	4 .19	Naive	SOF+DCV (24)	SVR24	None	None
13	no	4	neg	4 .84	Naive	SOF+RBV	n.a.	A62P	None

						(40)	***		
14	no	4	neg	5 .34	Experienced	SOF+RBV (37)	SVR24	None	None
15	no	4	neg	4 .78	Experienced	SOF+RBV (24)	SVR24	A30K	None
16	no	4	neg	3 .19	Naive	SOF+pIFN+RBV (12)	SVR24	n.d.	None
17	no	4	neg	1 .08	Experienced	SOF+DCV (24)	SVR12	None	None
18	no	4	neg	6 .31	Experienced	SOF+DCV+RBV (24)	SVR12	None	None
19	no	4	neg	5 .10	Experienced	SOF+DCV+RBV (24)	SVR12	A30K	None
20	no	4	neg	4 .37	Experienced	SOF+DCV+RBV (24)	SVR12	None	None
21	no	4	neg	6 .63	Naive	SOF+DCV+RBV (24)	SVR24	None	None
22	no	4	neg	4 .61	Naive	SOF+DCV+RBV (24)	SVR12	None	None
23	no	4	neg	6 .58	Naive	SOF+DCV+RBV (24)	SVR24	A62T	None
24	no	3	pos	6 .19	Experienced	SOF+DCV+RBV (12)	SVR12	None	None
25	no	4	neg	4 .83	Experienced	SOF+DCV+RBV (24)	SVR24	None	None
26	no	4	neg	5 .30	Naive	SOF+DCV+RBV (24)	SVR24	None	None
27	no	4	neg	5 .14	Experienced	SOF+DCV+RBV (24)	SVR24	None	N244D
28	no	4	neg	6 .84	Experienced	SOF+DCV+RBV (24)	SVR24	A30K Y93N Y93D	None
29	no	4	neg	5 .74	Experienced	SOF+RBV (24)	SVR24	None	n.d.
30	yes	3	neg	5 .05	Naive	SOF+DCV+RBV (24)	SVR24	None	None
31	no	4	neg	5 .82	Naive	SOF+RBV (24)	SVR24	None	N244D N310D
32	no	4	neg	4 .76	Naive	SOF+DCV+RBV (24)	SVR24	None	N244D N310D
33	no	2	pos	6 .04	Experienced	SOF+RBV (24)	SVR24	None	None
34	yes	2	neg	5 .41	Naive	SOF+DCV (24)	SVR12	None	None
35	yes	1	neg	4 .03	Naive	SOF+DCV+RBV	SVR12	A62T	None

						(24)			
36	no	4	neg	5 .84	Naive	SOF+DCV+RBV--> SOF+RBV (8 wk) (36)	SVR24	None	None
37	n.k.	n.k.	neg	5 .40	n.k.	n.k.	n.k.	None	N310D
38	yes	2	neg	6 .68	Naive	SOF+RBV (24)	SVR24	None	None
39	yes	1	neg	6 .40	Naive	SOF+RBV (24)	SVR24	None	n.d.
40	no	4	neg	3 .82	Naive	SOF+RBV (20)	SVR24	n.d.	None
41	no	4	neg	5 .95	Experienced	SOF+pIFN+RBV (12)	SVR24	n.d.	None
42	yes	3	neg	6 .96	Naive	SOF+DCV (12)	Relapser	A30K	None
43	no	4	neg	4 .97	Naive	SOF+DCV (24)	BT	P58S	None
44	no	4	neg	5 .95	Experienced	SOF+RBV (24)	Relapser	M28L	None
45	no	4	pos	4 .58	Experienced	SOF+RBV (24)	Relapser	None	None

Pt=patient; LT=liver transplantation; P-R=pegylated interferon (pegIFN)+ribavirin (RBV); BT= breakthrough; n.a.=not available; n.k.=not known; n.d.=not determined; SVR12=sustained virological response at week 12; SVR24=sustained virological response at week 24; DAA= direct antiviral agent; wks=weeks; SOF=sofosbuvir; DCV=daclatasvir; RAS=resistance-associated substitution; BT= breakthrough.

* Diagnosis of liver cirrhosis was based on clinical or histological features or with non-invasive assessment by transient elastography (stiffness >14 KPa).

**On the basis of last information available at the time of writing the paper.

***This patient died just after having undetectable HCV RNA at the end of treatment.
